# Household Costs of Healthcare during Pregnancy, Delivery, and the Postpartum Period: A Case Study from Matlab, Bangladesh

**Published:** 2006-12

**Authors:** Josephine Borghi, Nazme Sabina, Lauren S. Blum, Mohammad Enamul Hoque, Carine Ronsmans

**Affiliations:** ^1^ Infectious Disease and Epidemiology Unit, London School of Hygiene & Tropical Medicine, London, UK; ^2^ International Perinatal Care Unit, Institute of Child Health, University College London, London; ^3^ ICDDR,B, GPO Box 128, Dhaka 1000, Bangladesh

**Keywords:** Healthcare costs, Health expenditure, Pregnancy, Childbirth, Health equity, Retrospective studies, Bangladesh

## Abstract

A household survey was undertaken in Matlab, a rural area of Bangladesh, to estimate the costs incurred during pregnancy, delivery, and the postpartum period for women delivering at home and in a health facility. Those interviewed included 121 women who delivered at home, 120 who delivered in an ICDDR,B basic obstetric care (BEOC) facility, 27 who delivered in a public comprehensive obstetric care (CEOC) hospital, and 58 who delivered in private hospitals. There was no significant difference in total costs incurred by those delivering at home and those delivering in a BEOC facility. Costs for those delivering in CEOC facilities were over nine times greater than for those delivering in BEOC facilities. Costs of care during delivery were predominant. Antenatal and postnatal care added between 7% and 30% to the total cost. Services were more equitable at home and in a BEOC facility compared to services provided at CEOC facilities. The study highlights the regressive nature of the financing of CEOC services and the need for a financing strategy that covers both the costs of referral and BEOC care for those in need.

## INTRODUCTION

The health of women around the time of delivery remains a major concern in Bangladesh where the ratio of maternal mortality is over 322 per 100,000 (1). Skilled attendance at delivery has been promoted as the single most effective means of successfully reducing rates of maternal mortality in poorer countries (2). Yet, in Bangladesh, the majority of mothers do not use skilled delivery care due to a combination of sociocultural barriers (3) and issues associated with the availability, quality, and cost of services (4, 5). While 91% of deliveries take place at home, a trained health worker is present in only 13% of cases, with most deliveries being attended either by relatives or by a traditional birth attendant (TBA) (6).

Financial cost plays an important part in the demand for healthcare in general (7) and for maternity care in particular (8–10)). A number of studies have estimated the household costs of antenatal (11, 12) and obstetric care (13–14). These studies have focused mainly on the medical costs incurred within facilities and, in some cases, transport costs. Little has been reported about costs incurred by those delivering at home. Comparing costs of facility and home-based professional care is particularly relevant in settings, such as Bangladesh, where birth at home with a skilled attendant forms an essential part of the national safe-motherhood strategy (16). Furthermore, little is known about the equity implications or affordability of such cases. The cost—both financial and time—incurred by companions accompanying delivering mothers to a health facility is a further component of cost that has received minimal attention. Generally, seeking care in a facility requires women to be accompanied by a family member, particularly in societies where restrictions are placed on female mobility. Lastly, the studies to date have considered the cost of specific services, e.g. antenatal or delivery care, without estimating the total costs incurred during pregnancy, delivery, and the postpartum period. Despite the lack of studies in this area, the cumulative cost of pregnancy, delivery, and postpartum care is potentially significant.

Against this background, we conducted a household survey in rural Bangladesh to estimate the overall costs incurred during pregnancy, delivery, and the postpartum period by place of delivery (at home or in a health facility) and by wealth group.

## MATERIALS AND METHODS

### Study site

Since 1966, ICDDR,B has maintained a registration of all births, deaths, and migrations in Matlab, a rural area of Bangladesh. Facilities within the area are funded, staffed, and maintained by ICDDR,B and provided free of charge.

Within this area, deliveries at home with skilled attendants, who were either midwives or paramedics, were advocated from 1987 to 1996, and from 1996 onwards, maternity care in basic obstetric facilities was gradually phased in. To this end, four existing health centres were upgraded with a delivery room, and the same healthcare workers, who had been working in homes, shifted to facility-based care, catering mostly for uncomplicated deliveries. A basic essential obstetric care (BEOC) hospital was also set in place. These facilities are within three kilometres of most households.

Complicated deliveries can be managed at comprehensive essential obstetric care (CEOC) facilities either at the government district hospital in Chandpur or at private hospitals, of which there are a growing number. These facilities provide, and charge for, essential obstetric care, conduct caesarean sections, and perform assisted deliveries by forceps and vacuum. A free ambulance service takes women from the BEOC facility to the CEOC facility.

### Identification of costs

Two questionnaires were designed—one for each place of delivery (home and health facility)—to collect information on all monetary expenditure made by household members to access and receive antenatal care, delivery, and postnatal care. The cost of services provided by traditional practitioners was also estimated. Transport costs to and from the health facility were estimated for all types of care. For delivery care, additional categories, such as medical costs and costs of food, cleaning materials, and service tips, were considered. Medical costs at home included supplies, such as mustard oil, safe delivery-kits, and drugs; and in the health facility, the medical costs included drugs, laboratory tests, and any other medical supplies. Women were asked to estimate the monetary costs involved in preparing special food for the woman giving birth. For women delivering in the health facilities, food was brought in from outside by family members. For women delivering at home, the costs were adjusted to match the time spent in a BEOC facility, for comparability. Costs associated with cleaning were also estimated as cleaning materials were often used for cleaning up after the delivery, and the household would generally pay someone to carry out this procedure. For all other services accessed during pregnancy and the postpartum period, data were collected on the total cost of consultation and the cost of drugs and medical supplies.

As time and monetary factors can delay access to obstetric care, information was collected on the time spent calling and waiting for attendants (for delivery at home) and also for travelling to facilities and waiting for treatment. Respondents were questioned about the occupation of companions and whether they had lost any income or incurred any expenditure during their stay at the health facility, such as for transport, accommodation, or food. The financial and time cost data of companions were not collected for antenatal or postnatal care services.

To explore equity issues, the socioeconomic status of households was measured using a wealth index based on asset ownership (17), which was calculated for the Matlab area as a whole in 1996 (18). The original index was measured in quintiles, but was adjusted to terciles to provide groups of similar size for our study sample. Health expenditure has been defined as catastrophic if it exceeds 10% of the annual household income remaining after subsistence needs have been met (19). Due to difficulties in measurement, information on the income of husbands rather than household income was obtained. In this study, costs were considered to be catastrophic if they exceeded 20% of the annual income of a husband. Questionnaires were translated into Bangla and back-translated into English. Four female data collectors were trained on the use of the questionnaires during a one-week session. The research team piloted the questionnaires, and changes were made accordingly.

### Sample

We sampled for the following categories of obstetric care: delivery at home with a TBA, delivery at home with a midwife, delivery in a BEOC facility, and delivery in two types of CEOC facility (government district hospital and private clinics). As this was a retrospective study relying on household recall, efforts were made to interview those who had most recently delivered. To validate the data obtained, the field researchers cross-checked with other household members where possible. For institutional deliveries, facility-based costs were verified in the facilities, and prices of drug and laboratory tests were verified in drug stores and diagnostic centres. For other costs, such as gifts, food, and cleaning materials, we relied on the memory of respondents and household members.

Those who delivered at home or in a BEOC facility were identified from the demographic database of all households starting from births taking place in 2002 and working backwards through time until 260 births had been identified. In some areas, women began delivering at health centres from 1996. Therefore, some of those interviewed for delivery at home with a midwife actually gave birth in 1995. As there was no significant difference between the costs reported at that time and the costs reported for deliveries in 2001, we did not adjust costs for inflation.

The sample size was pragmatically determined based on available time and resources. Those who were referred from a Matlab BEOC facility and delivered in a CEOC facility were identified from a list compiled by researchers examining the extent of ‘unmet’ need for obstetric care for women who gave birth between 2001 and 2002. The list indicated the type of delivery and household identity number. Fifty-eight women who gave birth in a private facility were selected for interview. Most of these women had a caesarean section. As much fewer women were referred to a public CEOC facility, only 27 such women were interviewed. These were a mix of caesarean section and vaginal deliveries. Interviews were conducted between February and November 2003.

### Analysis

All costs are presented in Taka (US$ 1=Tk 57.5 in 2002). To value the time of companions, their occupations and average daily wages were identified. Data were double-entered and analyzed using SPSS (version 12). Means (95% confidence interval) and medians (25th and 75th percentiles) were used for indicating the average costs, and confidence intervals and inter-quartile ranges were used for illustrating the extent of variation within the sample. Statistical significance was measured by the Mann-Whitney U test and *t*-test for non-normal and normally distributed continuous variables respectively.

## RESULTS

### Description of sample

We interviewed 121 women who delivered at home: 61 were attended by a midwife, and 60 were attended by a TBA. We interviewed 120 women delivering in a BEOC facility, 61 in a health centre, and 59 in the BEOC hospital. We interviewed 27 women delivering in a district hospital and 58 women delivering in a private clinic. Ten of the 27 deliveries in the district hospital and 55 of the 58 deliveries in the private facilities were caesarean sections. The remaining cases were vaginal births. The births took place between 1995 and 2002. The recall period was longest for births at home, at mean 3.2 (median 1.1) years for those delivering with the assistance of a TBA and at 4.8 (median 4.0) years for those delivering with the assistance of a midwife. For facility-based deliveries, the recall period ranged from mean 1.5 (median 1.4) years for those delivering at BEOC facilities and 2.3 (median 2.4) years for those delivering at CEOC facilities.

Sixty percent of those interviewed, who had delivered at home, had either primary education or no education. Over half of those delivering in a health facility had secondary-level education or above. There was no significant difference in the average monthly income of husbands of those women delivering at home, in a BEOC facility, or in a public CEOC facility; the median income was Tk 3,675 (range Tk 2,100–6,000). The average monthly income of husbands whose wives delivered in a private facility was significantly higher than the others; the median income was Tk 8,000 (Tk 4,300–12,000) (p<0.01). Significantly more households delivering privately were in the least poor group compared to households delivering elsewhere (56% vs 28% respectively) and significantly less were in the most poor group (12% vs 34%) (p<0.01). The average monthly income of husbands for the poorest group was Tk 3,514 (median Tk 2,670), increasing to Tk 4,721 (median Tk 3,950) for the middle group, and to Tk 9,877 (median Tk 5,000) for the least poor group.

### Time and monetary cost of obstetric care

There was no significant difference in expenditure for deliveries at home attended either by a midwife or by a TBA (median Tk 135 and Tk 184 respectively) (Table 1). The costs in a health centre were significantly more than those at home if companion costs (both financial and time-related) were included (p<0.05). When companion costs were not included, the difference between the costs of those delivering at home and those in a health centre were not significant. Those who delivered at the Matlab hospital paid significantly more than those who delivered at home (p<0.01) (Table 2). Household expenditure on medical/non-medical supplies and tips was significantly greater for those delivering at home compared to those delivering at a health centre (p<0.05). However, overall, those who delivered in a BEOC facility spent significantly more money on food than those who delivered at home.

**Table 1.** Total costs (Tk) of delivery at home

Cost item	Delivery at home			
	TBA (n=60)		Midwife (n=61)	
	Mean (95% CI)	Median (25^th^–75^th^ percentiles)	Mean (95% CI)	Median (25^th^–75^th^ percentiles)
Transport	0	0	0	0
Medical	90 (59–122)	58 (25–104)	84 (60–108)	50 (17–120)
Cleaning	19 (3–35)	0 (0–13)	13 (4–22)	0 (0–15)
Food	88 (4–172)	25 (1–52)	27 (17–37)	15 (0–40)
Tips	134 (90–178)	50 (0–250)	148 (62–234)	0 (0–235)
Total	331 (211–451)	184 (88–396)	271 (177–366)	135 (50–338)

CI=Confidence interval;

TBA=Traditional birth attendant

**Table 2.** Total costs (Tk) of delivery in a health facility

Cost item	Basic essential obstetric care facility				Comprehensive essential obstetric care facility			
	Health Centre (n=61)		Hospital (n=59)		Public (n=27)		Private (n=58)	
	Mean (95% CI)	Median (25^th^–75^th^)	Mean (95% CI)	Median (25^th^–75^th^)	Mean (95% CI)	Median (25^th^–75^th^)	Mean (95% CI)	Median (25^th^–75^th^)
Transport	10 (6–14)	5 (0–15)	23 (20–27)	25 (10–35)	132 (47–216)	35 (0–144)	186 (121–252)	40 (0–515)
Medical	75 (33–117)	0 (0–78)	251 (5–496)	48 (27–138)	7,358 (5,201–9,515)	7,000 (3,575–10,000)	21,901 (19,656–24,146)	20,000 (16,000–25,175)
Cleaning	12 (5–19)	0 (0–19)	15 (6–25)	0 (0–14)	13 (4–22)	0 (0–27)	14 (1–26)	0 (0–0)
Food	171 (122–219)	110 (58–206)	265 (148–381)	120 (60–300)	757 (406–1,108)	345 (161–1,058)	840 (619–1,060)	570 (150–1,500)
Tips	52 (25–78)	0 (0–88)	58 (17–98)	0 (0–0)	333 (59–606)	0 (0–500)	399 (188–610)	175 (0–400)
Companion financial costs	110 (67–153)	40 (0–135)	270 (155–385)	115 (50–330)	568 (361–775)	410 (198–763)	905 (577–1,234)	600 (270–1,080)
Companion time costs	10 (3–19)	0 (0–0)	161 (35–287)	0 (0–84)	290 (-18–599)	0 (0–140)	216 (79–353)	0 (0–201)
Total	440 (338–543)	326 (105–655)	1,043 (614–1,472)	540 (295–1,030)	9,451 (6,640–12,261)	9,244 (4,327–12,740)	24,461 (22,049–26,796)	23,305 (18,335–29,771)

CI=Confidence interval

Over 80% of the costs incurred within CEOC facilities were medical expenses. Costs of private-sector emergency care were more than twice that of public facilities (p<0.01). The costs incurred for a normal delivery were approximately half that of a caesarean section in CEOC facilities: median of Tk 4,633 (range Tk 2,775–9,929) compared to Tk 10,130 (Tk 6,390–18,328) for a vaginal delivery and Tk 10,986 (Tk 8,699–14,303) compared to Tk 22,530 (Tk 18,434–28,838) for a caesarean section in public and private facilities respectively. All costs incurred at CEOC facilities were significantly higher compared to the costs incurred at BEOC facilities.

Calling a midwife for a delivery at home took significantly longer than calling a TBA: median one hour (30–75 minutes) versus median 15 minutes (10–30 minutes) (p<0.01); or travelling to a health centre: median 20 minutes (14–30 minutes) (p<0.01). There was no significant difference in time spent travelling to a BEOC compared to a CEOC facility (median one hour [30–165 minutes]). Of those interviewed, a median of three people (2–4 people) accompanied each woman to the facility. They stayed a median four hours (2–8 hours) at BEOC and 10 hours (5 to 23 hours) at CEOC facilities (including travel time to and from the facility). The total financial cost incurred by companions together varied from median Tk 80 (Tk 12–200) in a BEOC facility to Tk 500 (Tk 215–575) in a CEOC facility. Over 4% of those delivering at a BEOC facility and 12% of those delivering at a CEOC facility reported that their companions also lost income. For the whole sample, the mean total income loss to companions was Tk 85 (median Tk 0) in a BEOC facility and mean Tk 238 (median Tk 0) in a CEOC facility.

### Equity

For those delivering in a CEOC facility, there was no significant difference in the median expenditure incurred by those in the poorest groups compared to the least poor groups, adjusting for type of delivery (Table 3). For those delivering in a BEOC facility or at home, the difference between the amount paid by the poorest and the least poor was borderline significant (p<0.1). While the poorest paid significantly less on food and tips than the least poor households (p<0.05), the expenditure on medical and non-medical supplies did not differ by wealth group.

**Table 3.** Total costs (Tk) of delivery by wealth tercile*

Wealth tercile	Delivery at home				Delivery in BEOC facility		Delivery in CEOC facility	
	TBA		Midwife					
	Mean (95% CI)	Median (25^th^–75^th^)	Mean (95% CI)	Median (25^th^–75^th^)	Mean (95% CI)	Median (25^th^–75^th^)	Mean (95% CI)	Median (25^th^–75^th^)
Poorest	179 (78–280)	91 (43–295)	114 (68–160)	95 (41–190)	480 (108–852)	170 (83–321)	19,483 (11,565–27,401)	15,360 (9,910–28,250)
Poor	285 (163–406)	191 (100–363)	244 (149–340)	174 (59–351)	452 (284–620)	275 (124–653)	18,615 (14,163–23,068)	17,000 (8,465–27,738)
Least poor	617 (110–1,123)	320 (125–540)	529 (166–891)	266 (77–844)	457 (307–607)	280 (163–673)	18,624 (15,949–21,299)	20,125 (13,515–24,016)

*Does not include the cost of companion time or expenditure,

BEOC=Basic essential obstetric care;

CI=Confidence interval;

CEOC=Comprehensive essential obstetric care;

TBA=Traditional birth attendant

### Affordability

For those delivering at home or in a BEOC facility, the total cost equated to 2% of the annual income of their husbands with companion costs, or 1% without. For those delivering in a CEOC facility, the costs equated to 31–41% of the annual income of their husbands in the public and private facilities respectively. For a vaginal delivery in a CEOC facility, the cost equated to mean 18% (median 13%) of the annual income of the husband compared to mean 45% (median 30%) for a caesarean section.

While deliveries at home were completely funded from household income and savings (no debt was incurred), facility-based deliveries necessitated the sale of assets or a loan in 4% of deliveries in a BEOC facility and 11% of deliveries in a CEOC facility.

### Costs of antenatal and postnatal care

In addition to the expenditure during delivery, 62% of women incurred costs associated with care received either antenatally or postnatally. Antenatal care and postnatal care with an ICDDR,B midwife were most frequently reported, although a number of women also sought care from a TBA or a traditional practitioner or from private clinics. Those who sought care at a private facility or from a TBA or a traditional practitioner paid significantly more than those receiving care from the ICDDR,B midwives (p<0.01) (Table 4). In addition to the charging system, these higher costs may result from patients presenting with complications as 58% of these women subsequently gave birth in a CEOC facility. In addition, 89 of the 326 interviewed women and children were admitted/re-admitted to a health facility for the treatment of postpartum complications (18% of those women who had delivered at home and 36% who had delivered in a facility). There was no significant difference in the cost of postpartum admission whether it was for the mother or for the baby. Altogether, these services cost women a mean Tk 541 (Tk 364–718) and a median Tk 50 (Tk 0–392), in addition to the cost of obstetric care.

**Table 4.** Costs (Tk) incurred during antenatal and postpartum periods

Type of care	No.	Median no. of visits	Cost*					
			Transport		Care		Total	
			Mean (95% CI)	Median (25^th^–75^th^)	Mean (95% CI)	Median (25^th^–75^th^)	Mean (95% CI)	Median (25^th^–75^th^)
Antenatal care with midwife	207	3	45 (36–54)	20 (0–50)	51 (20–81)	0 (0–0)	96 (61–130)	30 (0–90)
Postnatal care with midwife	120	1	11 (8–15)	0 (0–20)	37 (7–67)	0 (0–0)	48 (18–79)	0 (0–30)
TBA/traditional practitioner	61	3	12 (0–25)	0 (0–0)	217 (16–417)	20 (0–155)	229 (18–439)	21 (0–165)
Outpatient private clinic	55	1	147 (101–194)	50 (20–240)	755 (442–1,068)	400 (200–800)	902 (559–1,245)	530 (300–1,000)
Postnatal inpatient admission	89	1	297 (-40–633)	40 (0–130)	711 (366–1,056)	100 (0–500)	1,051 (509–1,594)	200 (50–666)

*The cost figures are for the total number of visits throughout pregnancy;

CI=Confidence interval;

TBA=Traditional birth attendant

### Total cost of care during pregnancy and the postpartum

The figure presents the cost of each service purchased during pregnancy and the postpartum period as a percentage of the total cost, by place of delivery. It shows that, for all cases, obstetric care was the most costly procedure, although the other maternity services constituted between 7% and 30% of the total cost for those delivering in a CEOC facility and at home/in a BEOC facility respectively. The total direct costs (exclusive of companion costs) amounted to between just over Tk 325 (range Tk 138–618) per woman delivering at home and to Tk 379 (Tk 184–838) per woman delivering in a BEOC facility (Table 5). There was no significant difference in cost between those delivering at home with the assistance of a TBA or a midwife, or those in a BEOC facility.

**Fig.**
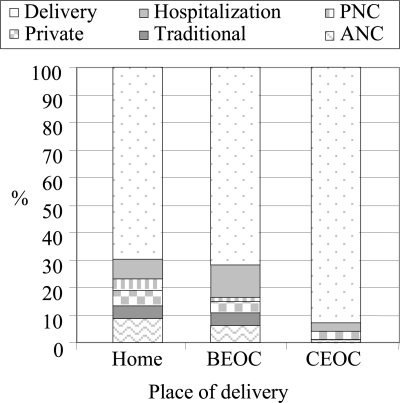
Components of pregnancy cost*

*Does not include the cost of companion time or expenditure
ANC=Antenatal care;
BEOC=Basic essential obstetric care;
CEOC=Comprehensive essential obstetric care;
PNC=Postnatal care

**Table 5.** Total cost (Tk) of pregnancy*

Place of delivery	No.	Mean (95% CI)	Median (25^th^–75^th^ percentiles)
Care with TBA at home	60	560 (347–772)	313 (156–644)
Care with midwife at home	61	533 (321–746)	325 (121–591)
BEOC facility	120	1024 (685–1364)	379 (184–838)
CEOC public hospital	27	9587 (6,724–12,450)	9220 (4,140–13,004)
CEOC private hospital	58	24,235 (21,967–26,503)	22,320 (18,248–29,300)

*This does not include the cost of companion time or expenditure;

BEOC=Basic essential obstetric care;

CEOC=Comprehensive essential obstetric care;

CI=Confidence interval;

TBA=Traditional birth attendant

The total cost of care during pregnancy, delivery, and the postpartum period amounted to mean 15% (median 9%) of the annual income of the husband of those delivering at home, increasing to 35% (median 12%) for those delivering in a BEOC facility. For those who delivered in a CEOC facility, the total pregnancy cost amounted to 452% (median 304%) of the annual income of their husband.

## DISCUSSION

The study illustrates a number of findings that are worthy of further discussion. The type of attendant (midwife or TBA) for deliveries at home had no significant impact on the expenditure incurred by households. However, the households indicated that it was quicker to call a TBA to their home to attend a delivery and, maybe more surprisingly, quicker for them to travel to a health centre than to call a midwife. This indicates that the number of midwives operating per population needs consideration when promoting a policy of deliveries at home with the assistance of midwives. Within the study area, there was one midwife per 6,000 women compared to one TBA per 80 women. Although the planned national midwife-to-population ratio is higher (one per 2,000 [http://w3.whosea.org/health_situt_98-00/annex1ban.htm, accessed on 9 July 2005]), midwives are still likely to take longer to reach households than TBAs who are in greater supply and proximity to households.

The difference in cost between delivery in a health centre and delivery at home was the costs incurred by companions. Both financial and opportunity costs to companions could, therefore, influence the decision to seek care outside the home, especially in societies where female mobility is more limited.

Overall, the costs incurred by those delivering at BEOC facilities was less than 10% of the annual income of their husbands and, therefore, did not reach catastrophic levels as defined by a 20% threshold. On the other hand, the cost to those delivering in a CEOC facility was well above the 20% threshold in the case of a caesarean section, representing almost half of the annual income of the husband. This is only the cost of delivery care, and once the costs of antenatal and postnatal services were added, the total increased substantially. The amount paid did not differ by wealth group but the immediate and longer-term financial burden of expenditure will most likely be greater for the poorest. This is consistent with findings of another study which states that, in Bangladesh, “the financing mechanisms were modestly regressive, and the distribution of government health expenditures was not pro-poor” (20). With the costs of medical care, such as drugs, forming the majority of the total bill in CEOC facilities, it is likely to be difficult for patients to negotiate the amount paid; exemptions do not appear to be effective. At home and in the BEOC facilities, where expenditure on discretionary items, such as food and tips, was predominant, households could contribute an amount based on their willingness and ability to pay.

The cost of a caesarean section was more than twice that of a vaginal delivery and, hence, the final bill administered to households was unpredictable, making it difficult for households to plan ahead. This is consistent with the findings reported elsewhere (14). Loans or a sale of assets were, therefore, required to finance care for a number of such households.

The consideration of the whole period of pregnancy, delivery and the postpartum gave insight into the extent of other maternity-related medical expenditure. Delivery care resulted in the greatest cost. Combined, the other maternity services accounted for 7–30% of the total cost.

This study had several limitations. Since it was carried out as part of an evaluation of policies which took place within two discrete time periods, we were obliged to interview women who had given birth within these periods. Consequently, our results depend upon the accuracy of household recall. As much as possible, interviews were conducted in the presence of husbands and mothers-in-law. The sample of women delivering in a public CEOC facility was small, although this reflected the current situation of low levels of service use.

The Matlab study area is distinctive compared to other parts of Bangladesh because of the nature of the BEOC facilities which are funded by ICDDR,B and do not charge (officially or unofficially) for services. However, this study demonstrates that the financing of care at lower levels should ideally be seen in conjunction with care at higher levels to offer real financial protection to households who need care at higher levels. Such an approach would help protect women from incurring high levels of expenditure. It would also most likely increase the use of healthcare with positive implications for mortality decline.

## ACKNOWLEDGEMENTS

This research was funded under the Cooperative Agreement No. 388-A-00-97-00032-00 with the United States Agency for International Development (USAID) (ICDDR,B Grant No. GR-00089). ICDDR,B acknowledges with gratitude the commitment of USAID to the Centre's research efforts. Carine Ronsmans and Jo Borghi are funded by the Department for International Development, UK. The authors are very grateful to Farhana Khanam for assisting in the undertaking of the study. They are also grateful for the hard work of Rowshan Ara Munni, Ayesha Siddika, Nazneen Rahman, Taniya Yesmin, and Sharmin Sultana Begum who collected data in the field.
